# Complete Genome Sequences of Six Copper-Resistant *Xanthomonas citri* pv. citri Strains Causing Asiatic Citrus Canker, Obtained Using Long-Read Technology

**DOI:** 10.1128/genomeA.00010-17

**Published:** 2017-03-23

**Authors:** Damien Richard, Claudine Boyer, Christian Vernière, Blanca I. Canteros, Pierre Lefeuvre, Olivier Pruvost

**Affiliations:** a Cirad, UMR PVBMT, St.-Pierre, Réunion, France; b ANSES, Plant Health Laboratory, St.-Pierre, Réunion, France; c Université de la Réunion, UMR PVBMT, St.-Denis, Réunion, France; d Cirad, UMR BGPI, Montpellier, France; e INTA, Estación Experimental Agropecuaria Bella Vista, Buenos Aires, Argentina

## Abstract

The gammaproteobacterium *Xanthomonas citri* pv*.* citri causes Asiatic citrus canker. Pathotype A strains have a broad host range, which includes most commercial citrus species, and they cause important economic losses worldwide. Control often relies on frequent copper sprays. We present here the complete genomes of six *X. citri* pv*. *citri copper-resistant strains.

Asiatic citrus canker, caused by *Xanthomonas citri* pv*. *citri, causes important economic losses in most tropical and subtropical areas. Its development impacts crop yields and also limits exports because of the quarantine status of the pathogen in some countries. The pathogen is currently classified into three pathotypes. Whereas pathotypes A^w^ and A^*^ have a restricted host range and geographical distribution, pathotype A has a wider host range, which has resulted in major geographical expansion of the pathogen (1). In the early stages of emergence, eradication is feasible through tree removal, burning, and quarantine restrictions, whereas integrated pest management (IPM) is conducted after the disease has spread in an area. IPM typically includes repetitive copper applications. Copper-resistant (Cu^R^) strains were first described in 1994 in Argentina (2), and 20 years later in the French islands of Réunion (3) and Martinique (4). To investigate the genetic basis of this resistance, six Cu^R^ strains were sequenced using long-read PacBio RSII technology with one single-molecule real-time (SMRT) cell per strain. Three strains originated from Réunion (LH201, LH276, and LJ207-7), two from Argentina (LM199 and LM180 = A44), and one from Martinique (LL074-4). Minisatellite typing revealed that all Cu^R^ strains were assigned to genetic lineage 1 within pathotype A (1).

The *de novo* genome assembly was conducted using the SMRT Analysis HGAP version 2.3 protocol. Circularization of the contigs was attempted using a combination of the Minimus assembler (5) and the SMRT Analysis resequencing version 1 protocol. The protein-coding sequences of the plasmid that confers Cu^R^ to LH201 were predicted using the MaGe genome annotation platform. In order to improve the assembly of transcription activator-like genes, additional assembly steps were performed in a manner similar to that as previously described (6).

The chromosomes of the six strains were fully reconstructed, with sizes ranging from 5,148,274 to 5,198,476 bp. Depending on the strains, two to four additional contigs were circularized into plasmids, with sizes ranging from 28,949 to 249,697 bp. All six strains hosted a plasmid carrying Cu^R^ genetic determinants. These plasmids were highly conserved among strains (despite remote geographical origins), apart from LM199. Whereas the other five strains hosted the *copLAB* gene system previously described for *Xanthomonas citri* pv. citri (7), LM199 encoded *copABCD*, which has already been described on the chromosome of *X. arboricola* pv. juglandis (8). Additional putative resistance gene clusters were found on the Cu^R^ plasmid: *cusAB/smmD*, *czcABCD*, and *arsBHCR*. However, LM199 lacked *czcABCD* and *arsBHCR*. MICs were evaluated as previously described (9): zinc chloride, 32 mg/L; copper sulfate, 128 to 256 mg/L; sodium arsenite, 8 to 16 mg/L (LM199), 64 mg/L (LH201), 128 mg/L (LL074-4), >128 mg/L (LH276, LM180, and LJ207-7); cadmium sulfate, 6.4 to 12.8 mg/L; and cobalt chloride 16 to 32 mg/L. Because strain LM199 (which did not contain the putative *czcABCD* region) and other sequenced strains (containing *czcABCD*) shared similar MIC values for cadmium sulfate, zinc chloride, and cobalt chloride, the function of this efflux pump remains unclear. These sequences highlight the potential of microbial adaptation through horizontal gene transfer and the mosaic structure of the genetic elements carrying adaptive genes.

## Accession number(s).

The six closed genome sequences have been deposited in GenBank. The genome accession numbers are summarized in Table 1.

**TABLE 1:** Characteristics of six *Xanthomonas citri* pv.* *citri strains

Strain	Other number(s)	Accession no.	Copper resistance location	Place of isolation	Yr of isolation	Host
LM199	*X. citri* pv. citri 15-4632 (INTA)^a^	MSQV00000000	Plasmid	Argentina	2015	Orange
LM180	*X. citri* pv. citri 03-1638 (INTA); A44^b^	MSQW00000000	Plasmid	Argentina	2003	Grapefruit
LL074-4		CP018847 to CP018849	Plasmid	Martinique	2014	Grapefruit
LJ207-7		CP018850 to CP018853	Plasmid	Réunion	2012	Kaffir lime
LH276		CP018854 to CP018857	Plasmid	Réunion	2010	Kaffir lime
LH201		CP018858 to CP018860	Plasmid	Réunion	2010	Kaffir lime

^a^ INTA, Instituto Nacional de Tecnología Agropecuaria.

^b^ As designated by Behlau et al. (7).

## References

[1] Pruvost O, Magne M, Boyer K, Leduc A, Tourterel C, Drevet C, Ravigné V, Gagnevin L, Guérin F, Chiroleu F, Koebnik R, Verdier V, Vernière C 2014 A MLVA genotyping scheme for global surveillance of the citrus pathogen *Xanthomonas citri* pv. *citri* suggests a worldwide geographical expansion of a single genetic lineage. PLoS One 9:e98129. doi:10.1371/journal.pone.0098129. 24897119 PMC4045669

[2] Canteros BI, Rybak M, Gochez A, Velazquez P, Rivadeneira M, Mitidieri M, Garran S, Zequeira L 2008 Occurrence of copper resistance in *Xanthomonas axonopodis* pv. *citri* in Argentina. Phytopathology 98:S30–S31.

[3] Richard D, Tribot N, Boyer C, Terville M, Boyer K, Javegny S, Roux-Cuvelier M, Pruvost O, Moreau A, Chabirand A, Vernière C 2017 First report of copper-resistant *Xanthomonas citri* pv. *citri* pathotype A causing Asiatic citrus canker in Réunion, France. Plant Dis [Epub ahead of print.] doi:10.1094/PDIS-09-16-1387-PDN.

[4] Richard D, Boyer C, Javegny S, Boyer K, Grygiel P, Pruvost O, Rioualec AL, Rakotobe V, Iotti J, Picard R, Vernière C, Audusseau C, François C, Olivier V, Moreau A, Chabirand A 2016 First report of *Xanthomonas citri* pv. *citri* pathotype A causing Asiatic citrus canker in Martinique, France. Plant Dis 100:1946. doi:10.1094/PDIS-02-16-0170-PDN.

[5] Sommer DD, Delcher AL, Salzberg SL, Pop M 2007 Minimus: a fast, lightweight genome assembler. BMC Bioinformatics 8:64. doi:10.1186/1471-2105-8-64. 17324286 PMC1821043

[6] Booher NJ, Carpenter SC, Sebra RP, Wang L, Salzberg SL, Leach JE, Bogdanove AJ 2015 Single molecule real-time sequencing of *Xanthomonas oryzae* genomes reveals a dynamic structure and complex TAL (transcription activator-like) effector gene relationships. Microb Genom 1:1–22. doi:10.1099/mgen.0.000032. PMC4853030 27148456

[7] Behlau F, Canteros BI, Minsavage GV, Jones JB, Graham JH 2011 Molecular characterization of copper resistance genes from *Xanthomonas citri* subsp. *citri* and *Xanthomonas alfalfae* subsp. *citrumelonis*. Appl Environ Microbiol 77:4089–4096. doi:10.1128/AEM.03043-10. 21515725 PMC3131652

[8] Pereira UP, Gouran H, Nascimento R, Adaskaveg JE, Goulart LR, Dandekar AM 2015 Complete genome sequence of *Xanthomonas arboricola* pv. juglandis 417, a copper-resistant strain isolated from *Juglans regia* L. Genome Announc 3(5):e01126-15. doi:10.1128/genomeA.01126-15. 26430043 PMC4591315

[9] Pruvost O, Couteau A, Perrier X, Luisetti J 1998 Phenotypic diversity of *Xanthomonas* sp.* mangiferaeindicae*. J Appl Microbiol 84:115–124. doi:10.1046/j.1365-2672.1997.00328.x. 15244066

